# The Enhancer Landscape during Early Neocortical Development Reveals Patterns of Dense Regulation and Co-option

**DOI:** 10.1371/journal.pgen.1003728

**Published:** 2013-08-29

**Authors:** Aaron M. Wenger, Shoa L. Clarke, James H. Notwell, Tisha Chung, Geetu Tuteja, Harendra Guturu, Bruce T. Schaar, Gill Bejerano

**Affiliations:** 1 Department of Computer Science, Stanford University, Stanford, California, United States of America; 2 Department of Genetics, Stanford University, Stanford, California, United States of America; 3 Department of Developmental Biology, Stanford University, Stanford, California, United States of America; 4 Department of Electrical Engineering, Stanford University, Stanford, California, United States of America; University of California San Francisco, United States of America

## Abstract

Genetic studies have identified a core set of transcription factors and target genes that control the development of the neocortex, the region of the human brain responsible for higher cognition. The specific regulatory interactions between these factors, many key upstream and downstream genes, and the enhancers that mediate all these interactions remain mostly uncharacterized. We perform p300 ChIP-seq to identify over 6,600 candidate enhancers active in the dorsal cerebral wall of embryonic day 14.5 (E14.5) mice. Over 95% of the peaks we measure are conserved to human. Eight of ten (80%) candidates tested using mouse transgenesis drive activity in restricted laminar patterns within the neocortex. GREAT based computational analysis reveals highly significant correlation with genes expressed at E14.5 in key areas for neocortex development, and allows the grouping of enhancers by known biological functions and pathways for further studies. We find that multiple genes are flanked by dozens of candidate enhancers each, including well-known key neocortical genes as well as suspected and novel genes. Nearly a quarter of our candidate enhancers are conserved well beyond mammals. Human and zebrafish regions orthologous to our candidate enhancers are shown to most often function in other aspects of central nervous system development. Finally, we find strong evidence that specific interspersed repeat families have contributed potentially key developmental enhancers via co-option. Our analysis expands the methodologies available for extracting the richness of information found in genome-wide functional maps.

## Author Summary

Sequencing based technologies provide global snapshots of transcriptional regulation. These data promise insights into gene regulation, disease susceptibility and organismal evolution. They also provide a methodological challenge in distilling specific hypotheses from large masses of data. Most work to date has focused on deriving broad biochemical insights. Here we obtain the active enhancer landscape of the dorsal cerebral wall during early neocortical development. We show that our set likely contains enhancers from both the developing neocortex, the ventricular, subventricular and intermediate zones, and develop methods to separate this mass into subsets of interest in particular contexts. We discover novel enhancers next to key neocortex development genes. We show that some known key and novel genes are regulated by dozens of enhancers each, and find known and novel enriched binding sites for key transcription factors in our set. Nearly all newly discovered enhancers are conserved in human. A quarter of loci are shared with non-mammalian vertebrates. We show that the human and zebrafish orthologs of our enhancers mostly drive expression in related nervous system contexts. We also show that particular interspersed repeats were preferentially co-opted into potentially key neocortex development enhancers.

## Introduction

**Figure 1:**
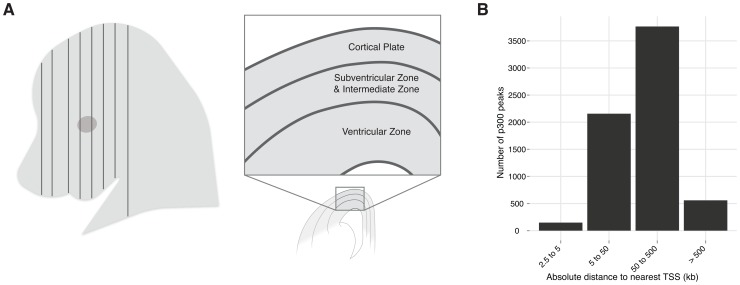
Neocortex development and evolution. A) A coronal plane of section through an embryo. One hemisphere is shown diagrammatically. The neocortex develops from the dorsal telencephalon. At E14.5 progenitor cells from the ventricular zone (VZ) are producing intermediate progenitor cells that migrate to form the subventricular and intermediate zones (SVZ-IZ); daughter cells from both areas migrate past the SVZ-IZ to form the cortical plate (CP), from which the neocortex develops (adapted from [7]). B) Absolute distance of the 6,629 p300 peaks (midpoint) to the canonical transcription start site of the nearest gene.

Among all vertebrates, the developing central nervous system segments into a forebrain, midbrain, hindbrain, and spinal cord [1]. The forebrain is further segmented into the telencephalon and diencephalon. In mammals, the dorsal portion of the telencephalon gives rise to the neocortex (isocortex). The mature neocortex is a complex six-layered structure unique to mammals [2], [3]. It has been associated with higher cognitive functions [4], and defects in this structure are the likely source for many neurologic and psychiatric diseases [5]. Early in development, this region consists of a layer of progenitor cells lining the ventricles called the ventricular zone (VZ). Progenitor cells of the VZ produce intermediate progenitor cells that migrate out of the VZ to form the subventricular and intermediate zones (SVZ-IZ); daughter cells from both areas migrate past the SVZ-IZ to form the laminar structure of the cortical plate (CP), in an inside out fashion [6], [7] (Figure 1A).

While the anatomy, histology, and gene expression patterns of the developing neocortex and its progenitor populations have all been well studied, attention is only starting to focus on gene regulation during neocortex development [8]. The advent of chromatin immunoprecipitation and related capture technologies, coupled with deep sequencing (ChIP-seq) allows us to obtain whole genome maps of active enhancers through development, and beyond. The study of enhancers provides several advantages: First, it reveals a sizable layer of genomic susceptibility to disease that extends beyond protein coding sequence, and has remained almost invisible hitherto. Second, because enhancers integrate signals from upstream transcription factors and signaling pathways, enhancer maps can unravel the causality of gene expression and developmental processes. Finally, observing enhancer sequence and function change between humans and related species promises to provide additional insights into the evolution of our brain.

Here, we produce an active enhancer map in the dorsal cerebral wall at E14.5 using ChIP-seq to assay for the enhancer-associated co-activator protein p300. We proceed to validate multiple enhancers next to genes of particular interest to neocortical development. We also develop a series of computational analyses that demonstrate the riches of information exposed by this type of assay for studies of neocortex development and evolution. Our methodology can be combined with current research in other tissues to advance our understanding of the complex regulatory networks that underlie organ development.

## Results

### E14.5 dorsal cerebral wall p300 ChIP-seq

To identify enhancers that function during neocortex development, we dissected the dorsal cerebral wall, which includes the developing neocortex and its progenitor populations, from E14.5 mouse embryos (Figure 1A) and performed chromatin immunoprecipitation followed by high-throughput sequencing (ChIP-seq) with an antibody against the enhancer-associated p300 co-activator complex (see Methods). This approach has successfully identified tissue specific developmental enhancers in several other contexts [9], [10]. We identified 6,629 p300 bound sites (>2.5 kb from the nearest transcription start site), which are candidate developmental enhancers (Table S1). As seen with other sets of enhancers [11], the majority of these elements are distal, with 65% being more than 50 kilobases from the nearest transcription start site (Figure 1B).

### Putative enhancer coherency with matched target genes expression

To globally assess the quality of our peak set, we first correlated the set with the pre-existing body of knowledge of neocortex development. Because p300 is an active enhancer mark, we asked whether our set of E14.5 p300 elements is correlated with gene expression patterns in the assayed tissue at the assayed time point.

GREAT (for Genomic Regions Enrichment of Annotations Tool) is an approach and web tool (at http://GREAT.stanford.edu/) devised specifically to assess enriched functions within a set of genomic regions thought to regulate the adjacent genes [11]. GREAT associates each gene in the genome with a variable length regulatory domain, bracketed by its two neighboring genes. GREAT holds a large body of knowledge about gene functions and phenotypes, curated from multiple different sources. Each term in GREAT is a list of genes that have functional commonalities (e.g. “involved in axon guidance”). Terms for a similar perspective of biology (e.g., molecular function) are collected into a GREAT ontology.

To quantify gene expression coherence we examined our set of p300 elements against the GREAT “MGI expression” ontology. This ontology is built from the MGI Gene Expression Database [12], and lists endogenous genes expressed in specific anatomical structures at specific developmental stages during mouse development, curated from the literature.

To test our p300 set of elements against the GREAT “MGI expression” ontology, GREAT iterates over 8,374 different tissue-timepoint combinations (terms) found in the MGI expression ontology, asking whether p300 elements are particularly enriched in the regulatory domains of genes of any particular term. For example, 1,226 genes in the human genome are annotated for “Theiler stage (TS) 22 cerebral cortex”, which corresponds to our tissue and timepoint of interest [13]. Their GREAT assigned regulatory domains cover 15.86% of the genome. Of the 6,629 p300 elements, 1,051 (15.86%) are expected in the regulatory domains of these 1,226 genes by chance, whereas 1,811 p300 elements, 1.72 times as many, are in fact observed (p-value: 9.5×10^−124^). GREAT shows similar strong enrichments for TS22 telencephalon and forebrain expressed genes (Table 1).

**Table 1:** Top GREAT enrichments for the E14.5 dorsal cerebral wall p300 ChIP-seq set.

Ontology	Term	P-value	Fold	p300 Peaks
MGI Expression	Theiler stage 22 telencephalon	6.33E-128	1.68	1,980
	Theiler stage 22 cerebral cortex	9.46E-124	1.72	1,811
	Theiler stage 24 nervous system	1.16E-122	1.73	1,756
	Theiler stage 19 forebrain	9.07E-121	1.61	2,131
	Theiler stage 22 forebrain	3.22E-119	1.61	2,096
Layer specificity (see Methods)	Specific to E14.5 ventricular zone	1.05E-25	2.99	126
	Specific to E14.5 subventricular zone	8.77E-25	3.39	101
	Specific to E14.5 cortical plate	1.79E-18	1.82	251
GO Molecular Function	Sequence-specific DNA binding RNA polymerase II transcription factor activity	7.57E-37	2.24	307
GO Biological Process	CNS neuron differentiation	1.49E-56	2.47	389
	Glial cell differentiation	3.34E-50	2.74	288
	Gliogenesis	1.82E-45	2.53	300
	Axon guidance	2.70E-43	2.10	418
	Telencephalon development	1.00E-42	2.13	397
Pathway Commons	Notch	8.14E-13	2.38	87
	Reelin signaling pathway	2.16E-09	2.25	68
	Netrin-mediated signaling events	4.11E-09	2.28	64
Mouse Phenotype	Abnormal neuron differentiation	5.61E-66	2.06	659
	Complete perinatal lethality	1.02E-64	2.14	590
	Abnormal nervous system tract	3.17E-58	2.32	453
	Abnormal brain commissure morphology	1.74E-57	2.48	396
	Abnormal forebrain development	1.90E-56	2.38	419

P-value and fold are for the binomial test. Theiler state 22 corresponds to E13.5–E15 [13].

At E14.5, the transient embryonic ventricular (VZ) and subventricular (SVZ) zones generate neurons that migrate across the intermediate zone (IZ) to the overlying cortical plate (CP), where they differentiate to form the neocortex. Because the tissue we measured contained all these areas, we wanted to know whether the different areas are well represented in our p300 set. To do so we utilized data from a recent study that used RNA-seq to measure expression levels in the VZ, SVZ-IZ, and CP at E14.5, obtained via laser capture microdissection (LCM) [14] (Figure 1A). First we note that p300 itself is expressed very similarly in all three regions: 10.83 RPKM (mean Reads Per exonic Kilobase per Million mapped reads) in the VZ, 11.05 in the SVZ-IZ and 9.11 in the CP; in the 23rd–24th percentile of all measured genes in all three regions. By comparing expression of all genes across the three regions we constructed three smaller lists of genes exclusively expressed in only one of these regions (see Methods). We then used GREAT to assess our p300 set enrichment next to these region-specific genes. The set is enriched against all three (p-value between 1.1×10^−25^ and 1.8×10^−18^), suggesting that the p300 set sampled the major regions of the E14.5 developing neocortex (Table 1).

### Comparison to related enhancer ChIP-seq datasets

A very recent publication reports 4,425 peaks from assaying p300 in E11.5 mouse forebrain, and 1,132 peaks from assaying a p300/CBP antibody in P0 mouse cortex [15]. CBP is a close paralog of p300 which plays a similar role in mediating active enhancer interactions. Of our 6,629 E14.5 peaks, 1,340 (20.21%) overlap the E11.5 set, and 235 (3.55%) peaks overlap the smaller P0 set of peaks. Both enrichments are highly significant, attesting to the quality of our set (uniform shuffling of our E14.5 peaks, fold 53.68 for E11.5 forebrain and fold 28.53 for P0 cortex), yet 5,153 (77.73%) of our E14.5 peaks are novel, overlapped by neither set.

Another publication assays CBP in E16.5 cortical neurons cultured for 7 days, before and after membrane depolarization [16]. They obtain fewer than 1,000 peaks before and approximately 28,000 peaks after stimulation, the latter mostly subsuming the peaks pre-stimulation. Of our 6,629 E14.5 peaks, 2,187 (32.99%) are overlapped by the larger set. This overlap is also highly significant (uniform shuffling of our E14.5 peaks, fold 15.09), while 4,442 (67.01%) of our peaks are unique.

### Characterization of novel E14.5 neocortical enhancers

**Figure 2:**
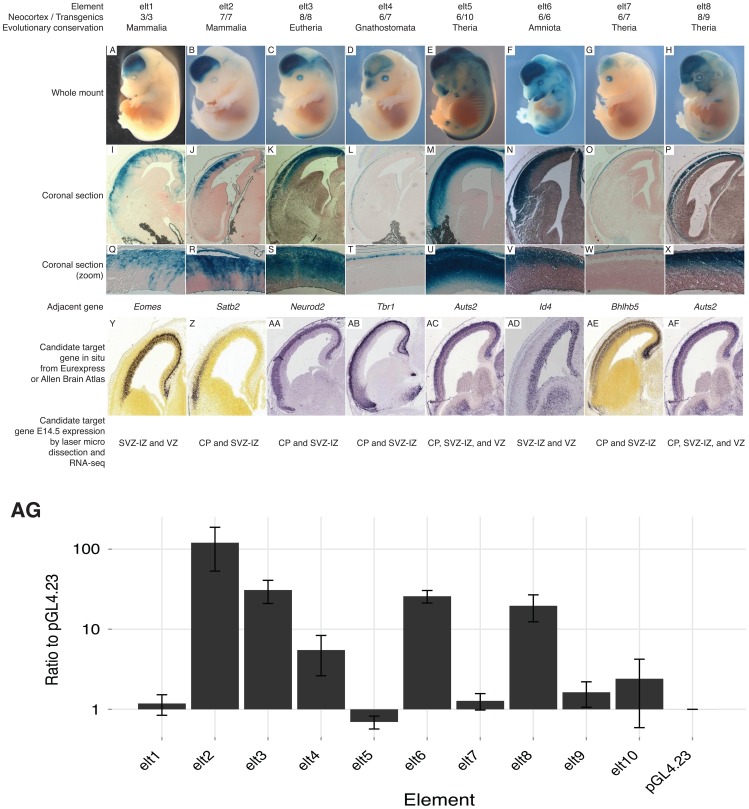
Candidate enhancers drive laminar expression in the developing mouse neocortex. Of the 10 assayed candidates, 8 (80%) drive reproducible expression in the developing neocortex. A–H) Whole mounts show expression in the cerebral cortex. I–P) Coronal sections reveal dorsal-specific expression exclusive of the ganglionic eminences. Q–X) Zooms of coronal sections reveal distinct laminar patterns. Y–AF) *In situs* of key neocortical genes found next to tested elements at E14.5 (coronal: Y–Z, sagittal: AA–AF). Y,Z,AE from Allen Brain Atlas; AA–AD,AF from Eurexpress. Below each gene *in situ* is the gene expression pattern from [14]. AG) Transfection results of our 10 elements in a higher throughput dissociated neuron transfection system. Five (63%) of eight transient transgenic positive enhancers drive high expression levels compared to the empty vector, and two transgenic negatives.

Previous studies of p300 ChIP-seq sets report up to 80% success in validating enhancer candidates using a transient transgenesis approach [9], [10], [17]. We chose ten enhancer candidates from our E14.5 p300 set, which lie next to genes known or suspected to play a role during embryonic neocortical development. None of these enhancer candidates overlapped a p300 peak from previous E11.5 forebrain (including both dorsal and ventral telencephalon) or P0 data [9], [15], and none have been reportedly previously tested in the VISTA browser [18]. Eight (80%) of these ten E14.5 p300 peaks drive reproducible expression in the developing neocortex in at least 3, and always a majority of positive embryos (Figure 2A–H; Figures S1, S2, S3, S4, S5, S6, S7, S8). Coronal sections reveal that the assayed enhancers drive dorsal-specific expression, exclusive of the ganglionic eminences of the ventral telencephalon (Figure 2I–P). Sections also reveal laminar restriction of enhancer activity (Figure 2Q–X, see Discussion).

Transient transgenesis experiments are low throughput and costly. To provide a higher-throughput cost-effective assay we also tested our ten candidates in a transient transfection system, where the dorsal cerebral wall is dissected and dissociated from the brains of E14.5 mice and then left to incubate for two additional days along with the transfected reporter constructs (see Methods). Five (63%) of the eight positive transgenics scored significantly higher than our empty vector and two negative transgenics in our transfection system (Figure 2AG). This suggests that our transient transfection system can provide a reliable, if imperfect, rapid system for preliminary screening of candidate developmental enhancers.

### The different functions regulated by the p300 enhancers

Our set of over 6,000 candidate enhancers likely regulates multiple different developmental processes that are taking place in the dorsal cerebral wall at E14.5. We use additional GREAT ontologies to parse out multiple different functions (Table 1): Using the Gene Ontology (GO) Molecular Functions ontology we see that our highest enrichment is for regulation of genes that themselves are involved in gene regulation (307 enhancers, p-value: 7.5×10^−37^), such as *Fox*, *Sox* and *Pax* transcription factors. The GO Biological Processes ontology highlights candidate enhancer groups that regulate processes well known to take place during neocortex development, including gliogenesis, axon guidance, and general telencephalon development. The Pathway Commons ontology highlights enhancer groups regulating specific pathways, including Notch, Reelin and netrin. The Mouse Phenotype ontology allows one to focus on groups of enhancers that regulate genes that share common cortical developmental defects, including abnormal neuron differentiation, abnormal forebrain development, and abnormal brain commissure development (Table 1).

### Enriched transcription factor regulators

ChIP-seq of different transcription factors (TFs) in a variety of contexts has shown them to bind reproducibly next to thousands of target genes. In particular, TFs have been repeatedly shown to bind near hundreds of genes specific to the contexts they are known to regulate, suggesting a high “fan out” of transcription regulation [11]. To search for some of the most abundant transcription factor binding motifs in our p300 set, we employed a standard three phase approach: First, we ran several published motif discovery tools to search *de novo* for over abundant motifs in our data; the obtained motifs were then compared to our library of known TF motifs to collapse redundant motifs; finally, the combined set of known and putative novel TF motifs were predicted across the p300 set and assessed for over-abundance against GC-matched control regions from the mouse genome (see Methods).

**Figure 3:**
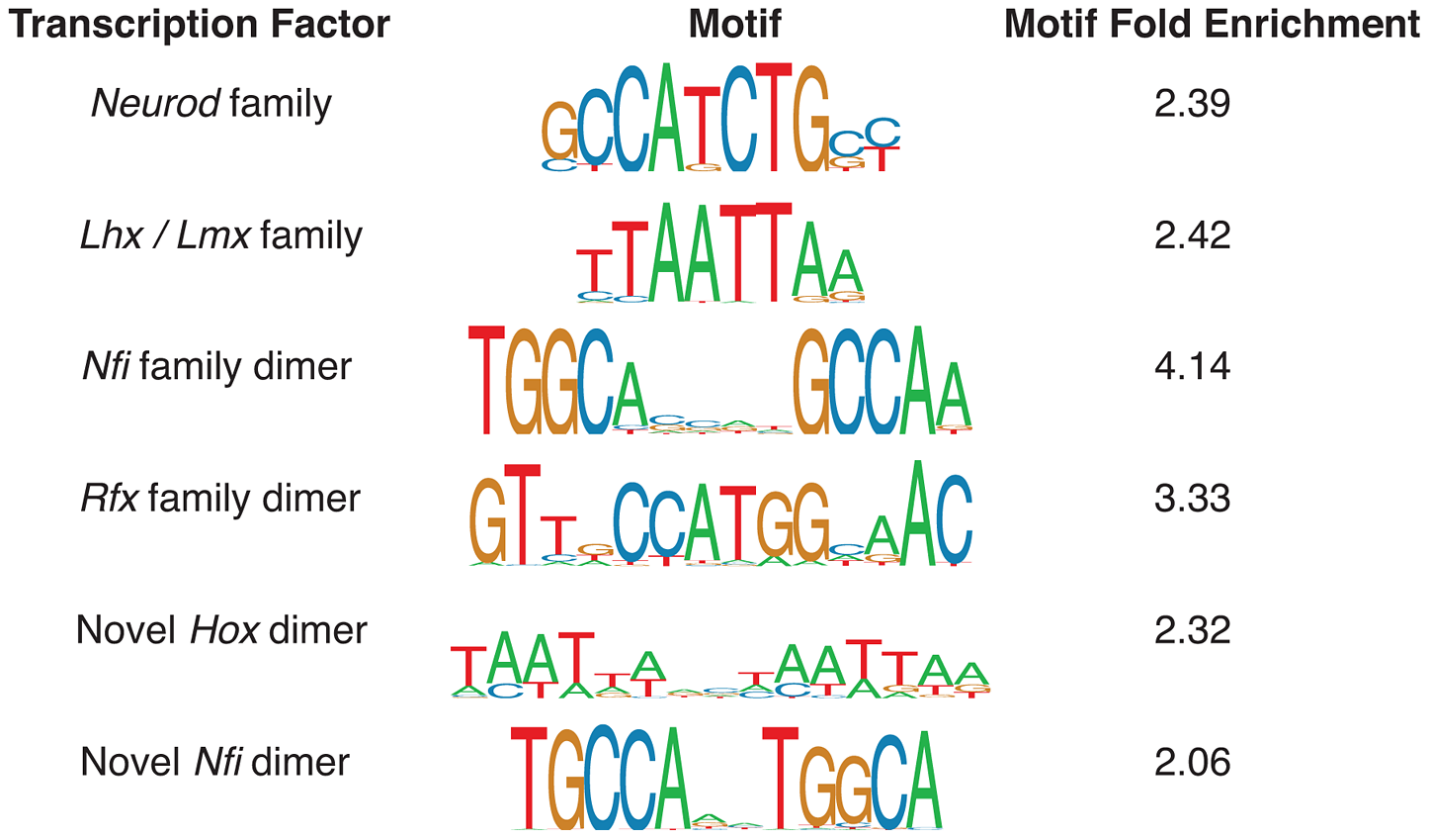
Monomer and dimer transcription factor motif predictions most enriched in the E14.5 p300 ChIP-seq set. Motif fold enrichment is relative to length and GC-matched regions of the mouse genome.

We identified a number of distinct enriched motifs, most of which belong to known important regulators of neocortex development (Figure 3). The *Neurod*/*Neurog* (2,452/6,629 enhancers = 37%; fold: 2.39), *Lhx*/*Lmx* (2,129 = 32%; fold: 2.42), *Nfi* (325 = 5%; fold: 4.14), and *Rfx* dimer (195 = 3%; fold: 3.33) motifs are all highly enriched in the candidate p300 enhancers. Factors from all four families have known roles in mammalian brain development [7], [19]–[21]. We also discovered two novel motifs enriched in the set: an alternative configuration from the known *Nfi* dimer motif [22] (379 = 6%; fold: 2.06) and a novel *Hox* dimer motif (473 = 7%; fold: 2.32).

### The most heavily regulated genes in the dorsal cerebral wall

**Figure 4:**
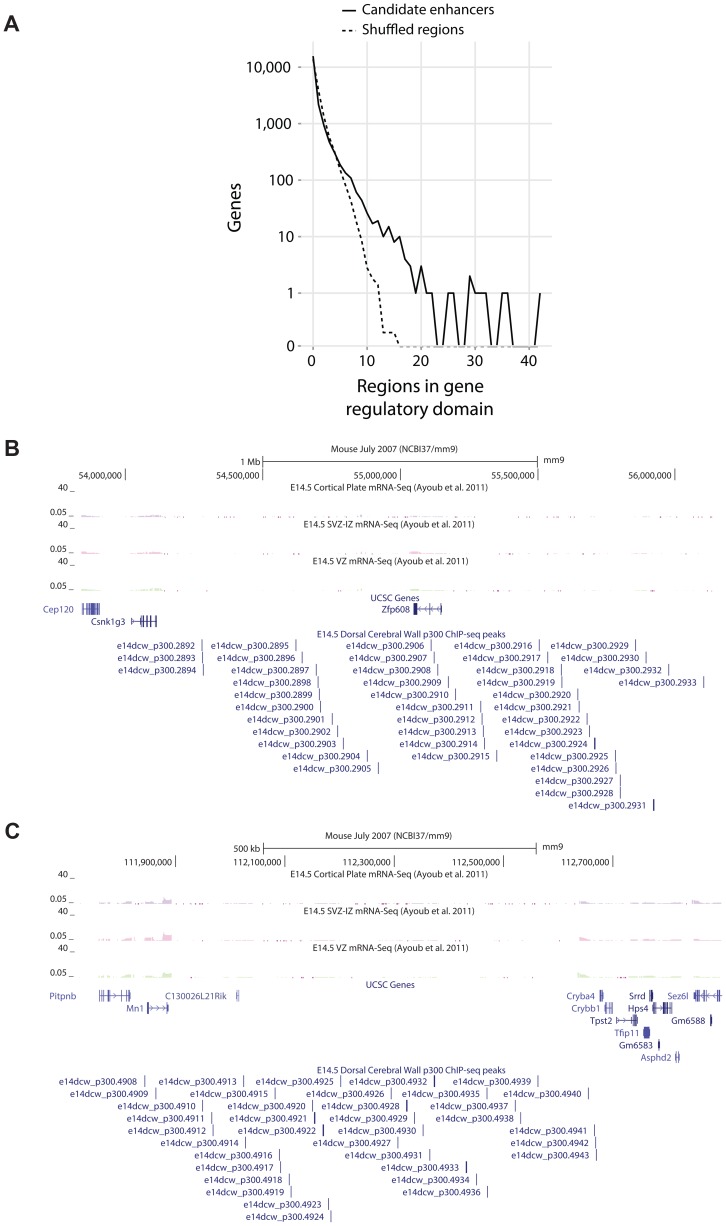
A) Observed number of candidate enhancers in the regulatory domain of all genes compared to random expectation. The top ten observed genes are listed in Table 2. B,C) Heavily p300 occupied putative gene regulatory domains around *Zfp608* and *Mn1*-*Cryba4*, respectively.

The candidate enhancers we measured exhibit a tendency to cluster together, with some genes having tens of p300 peaks in their predicted regulatory domains. To determine what would be expected by chance, we randomly distributed the 6,629 peaks across the genome 1,000 times. In this random null (which controls for gene regulatory domain length), we never observed any gene associated with more than 15 peaks (Figure 4A). In our true set, the most heavily regulated genes are associated with 20–42 peaks each. We can also use GREAT to rank all genes in the genome for the likelihood associated with the observed number of enhancers per gene vs. the length of the individual gene's regulatory domain (note that in this test, a gene with a smaller regulatory domain containing multiple enhancers, can rank higher than a gene with a much larger regulatory domain which contains more enhancers). When this variant of the GREAT test is run, the top ten most significant genes are the same ten genes with the absolute largest number of observed enhancers (p-value between 1.3×10^−15^ and 1.6×10^−31^). Three of these genes, *Nfib*, *Sox4* and *Sox11* are already known to play key roles in forebrain development. Three other genes, *Zfp608* (Figure 4B), *Auts2* and *Tle3* have previously been noted for their specific neocortical expression patterns, though their roles in its development are not well understood. Intriguingly, two additional gene deserts, flanked by the gene pairs *Mn1*-*Cryba4* (Figure 4C) and *Gse1*-*Fam92b*, all with unknown roles in neocortex development, are also packed with p300 elements (Table 2).

**Table 2:** The ten genes most enriched for the abundance of p300 peaks in their GREAT gene regulatory domains.

Likely target gene	P300 Peaks	Neocortical role	Literature support
*Zfp608* *	42	Suspected	“Determining the function of SVZ–IZ–specific novel transcription factors (e.g., *Zfp608*, …) may explain whether they contribute to an independent specification program or an intermediary implementation of the protomap” [14].
*Mn1* - *Cryba4* *	36	Unknown	“*MN1* is a cofactor of retinoic acid receptor/retinoic x receptor (RAR/RXR)-mediated transcription” [63].
*Sox11*	32	Established	“Cortex-specific double deletion of *Sox4* and *Sox11* leads to the loss of *Fezf2* expression” [8].
*Sox4*	31	Established	“Cortex-specific double deletion of *Sox4* and *Sox11* leads to the loss of *Fezf2* expression” [8].
*Nfib*	30	Established	“In addition to regulating the development of midline glial populations, *Nfib* also regulates the expression of neuropilin 1 within the cingulate cortex” [64].
*Gse1* - *Fam92b*	29	Unknown	*Gse1* is a coiled-coil protein of unknown function.
*Auts2*	29	Suspected	“The present study identified *Auts2*, a frontal cortex marker gene linked to autism and mental retardation, as a direct target of *Tbr1* binding and activation” [42].
*Tle3*	25	Suspected	“*TLE1* and *TLE3* were expressed in dorsal (i.e., neocortical) and ventral (lateral ganglionic eminence) telencephalic germinal zones” [65].

In two cases, a gene desert rich in p300 regions is flanked by two poorly studied genes in the context of neocortical development. By examining gene function and expression, our literature support points to the flanking gene more likely regulated by the peaks.

* Shown in Figure 4.

### Evolutionary conservation of our candidate enhancers

**Figure 5:**
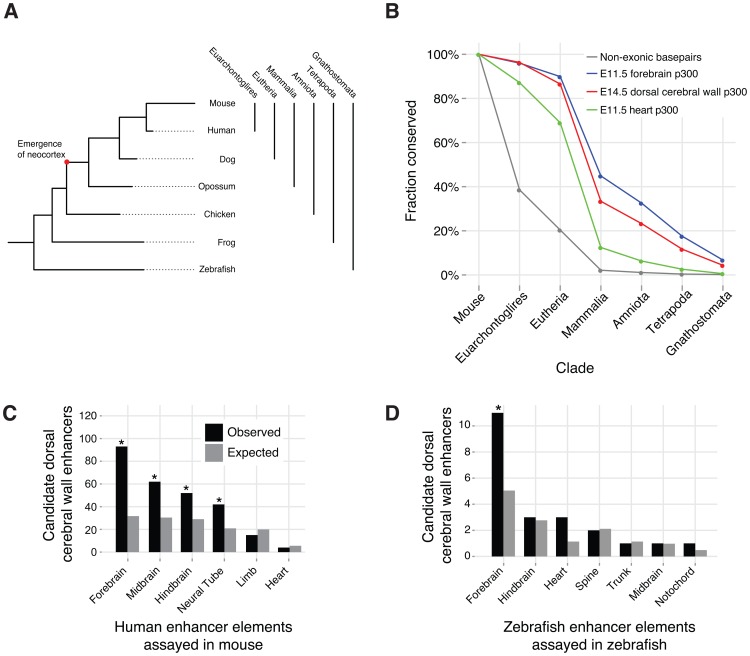
Conservation and additional functions of candidate dorsal cerebral wall enhancers. A) The phylogeny of vertebrate evolution. B) Evolutionary conservation of E14.5 dorsal cerebral wall enhancers compared to other p300 ChIP-seq sets and all genomic non-exonic bases. Each element belongs to a single x-axis category, which is the furthest evolutionary node to which it is conserved (see Methods). C) Overlap of E14.5 p300 peaks with regions for which the human ortholog functions in an E11.5 mouse transgenic enhancer assay (* denotes enrichment p-value <10^−5^). D) Overlap of E14.5 p300 peaks with regions for which the zebrafish ortholog functions in a zebrafish transgenic enhancer assay (* denotes enrichment p-value <0.05).

The six-layered neocortex is a mammalian specific innovation, while the progenitor populations are present in non-mammals [2], [3]. In non-mammalian jawed vertebrates (Gnathostomata in Figure 5A), the post-mitotic neurons do not organize into a six-layered cortex [3], [6]. In birds, for example, the neurons in the CP develop into the hyperpallium. Although the hyperpallium is topologically analogous to the neocortex, it has a nuclear structure rather than a laminar structure [3].

We examined cross species (orthologous) conservation of our 6,629 candidate enhancers to trace their origins and mode of evolution. The majority (4,278; 65%) of our candidate enhancers exhibit signatures of evolutionary sequence constraint (PhastCons score >350), suggesting that they have been evolving under purifying selection for millions of years. Very few elements appear specific to the mouse lineage. In particular, over 95% (6,317) are orthologously conserved to human. Over 86% (5,737) are common to all eutherian (placental) mammals. Nearly a quarter (1,543; 23%) of our peaks pre-date the mammalian innovation of the neocortex. In comparison, fewer than 5% of heart p300 ChIP-seq peaks [9] are conserved outside of mammals, and over 35% of forebrain p300 ChIP-seq peaks from E11.5 embryos [10] are conserved outside of mammals (Figure 5B). The forebrain encompasses both the telencephalon and diencephalon, and at E11.5 it consists of mostly progenitor cells [7]. The deeper conservation of E11.5 forebrain enhancers is consistent with the hypothesis that the early forebrain is more homologous across vertebrates [1].

### Dorsal cerebral wall enhancer function across different species

For 214 of our elements, the human ortholog has been tested in a mouse transgenic enhancer assay at E11.5 [23]. 148 of these elements function as developmental enhancers at this earlier time point. As expected, the majority of these elements indeed show expression in the forebrain. However, large and highly significant (all P<10^−5^, see Methods) subsets of active elements drive expression in additional structures of the developing central nervous system, including the midbrain, hindbrain and neural tube (Figure 5C).

Of our 6,629 p300 elements, 289 (4%) are conserved in fish. The zebrafish ortholog for 21 of our elements were assayed in a large zebrafish enhancer screen [24]. Twenty drive reproducible expression patterns in the developing zebrafish embryo. Again, the majority is seen to drive expression in the zebrafish forebrain (Figure 5D).

### *De novo* enhancer origin by co-option of interspersed repeats

Although a fraction of our candidate enhancers likely evolved from pre-existing enhancers (above), others have likely arisen *de novo* [25], [26]. One mechanism of particular interest for the generation of novel enhancers is through the co-option of mobile elements [27]–[29].

**Figure 6:**
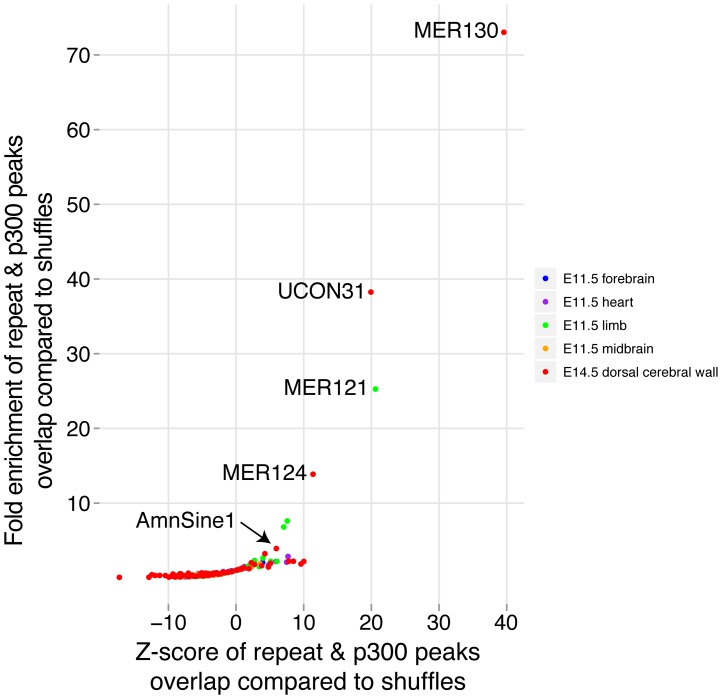
Co-option of mobile elements as dorsal cerebral wall enhancers. Each p300 ChIP-seq set was overlapped with all interspersed repeat families. For each combination, the expected number of overlaps was determined using 10,000 simulations where the p300 set was randomly distributed across the genome and overlaps were counted.

To determine if repetitive elements may have been co-opted as dorsal cerebral wall enhancers, we compared the overlap between our p300 set and all annotated interspersed repeat families in the UCSC genome browser. To control for the very different abundance of different repeat families, we shuffled our p300 set 10,000 times and noted the number of times the random sets overlapped each repeat family. For comparison, we repeated the same procedure with the four sets of previously obtained E11.5 p300 elements in forebrain, midbrain, limb and heart [9]. The most abundantly overlapping family of repeats with our E14.5 data is the MIRb family, which overlaps 238 p300 elements. This family has been noted before to be among the largest contributors to gene regulatory co-option among all mobile element families [30]. However, because many more copies of this repeat family are found in the genome, its fold enrichment of 1.84 against random overlaps is relatively low. In contrast, three poorly studied repeat families are found to make an extremely unlikely contribution to our p300 set: MER130, UCON31 and MER124. For the most enriched, MER130, 22 (24%) of 90 instances identified in the mouse genome overlap our E14.5 set, a 73 fold enrichment over expected (Figure 6).

### Enhancer function, origins, and phenotypic effect

**Figure 7:**
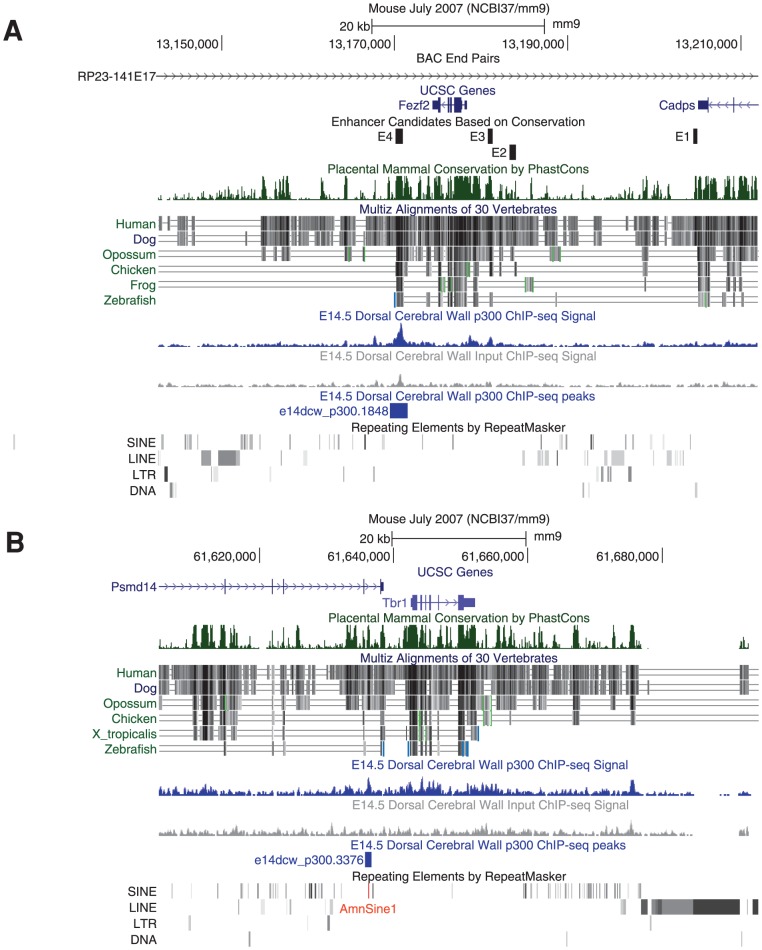
p300 peaks next to key neocortical developmental genes. A) A single E14.5 p300 peak (blue) is found in 180 kb of the RP23-141E17 BAC investigated in [8]. This peak contains the E4 developmental enhancer whose genomic deletion leads to aberrant cortico-spinal projection fates, similar to those found in its *Fezf2* target gene conditional knock-out. As suggested by our data, only deletion of E4, but not E1, E2 or E3 from the BAC resulted in reduced neocortical expression from the *Fezf2* locus. B) *Tbr1* and *Fezf2* act antagonistically to determine cortical neuron projection fates. A single E14.5 p300 peak is found proximal to the *Tbr1* gene. This pan-mammalian conserved peak has likely been seeded by the co-option of an AmnSine1 instance (red) at its center.

The p300 peaks we collected can at times be combined with signatures of genome evolution to accelerate functional analysis and hint at evolutionary developmental events of potential interest. For example, *Fezf2* is an important gene for neuronal fate determination. A recent paper studied the genomic regulation of *Fezf2* during neocortex development [8]. The authors first identified four sequence conserved genomic regions (dubbed E1–E4) flanking *Fezf2*. When each was separately deleted from a BAC containing a reporter gene knocked into the *Fezf2* gene locus – only E4 affected neocortical reporter gene expression. Impressively, the authors went on to show that a knockout of the E4 enhancer resulted in aberrant cortico-spinal projection, similar to mutant mice where the E4 target gene *Fezf2* has been deleted specifically in the cortex [8]. If we look at our data, E4 overlaps the one and only p300 peak observed in 180 kb of genomic sequence flanking the *Fezf2* locus in that BAC (Figure 7A).

During early neocortex development, *Fezf2* and *Tbr1* work in antagonistic fashion to determine different neuronal projection fates [31], [32], suggesting that a *Tbr1* regulatory element may play a similar key role to *Fezf2*'s E4. Downstream of *Tbr1* lies a 230 kb gene desert containing dozens of conserved elements, but completely devoid of our E14.5 p300 peaks. A single p300 element lies in the 50 kb upstream of *Tbr1*, 5 kb upstream of the gene, making it an intriguing candidate for further analysis (Figure 7B).

While the p300 peaks may currently serve to functionally pit *Fezf2* and *Tbr1* against each other, their evolutionary profile is markedly different. The *Fezf2* proximal p300 peak (E4) is conserved to fish, and does not overlap any known repeat. The human orthologous sequence of this peak drives forebrain expression in E11.5 transgenic mice [18], and the zebrafish orthologous sequence drives forebrain expression in 24-hour zebrafish embryos [24]. In contrast, the *Tbr1* peak is found only in mammals, and at its center lies a co-opted AmnSine1 repeat instance. The AmnSine1 repeat family is significantly enriched in our E14.5 set (3.9 fold, Figure 6). Intriguingly, of the 16 instances we observe overlapping our p300 set, four lie in the regulatory domains of genes that play crucial roles in neocortical neuron fate determination: *Tbr1* (above), *Satb2* (elt2 in Figure 2), *Sox5*, and *Reln*. Indeed, the *Satb2* co-opted element was recently characterized as a neocortex-specific enhancer [33].

## Discussion

In this study, we have identified the first genome-wide set of p300 bound regions specific to E14.5 dorsal cerebral wall. We have shown using GREAT and by sampling candidates experimentally that the set we obtained is highly enriched in active enhancers for neocortex development. This set of candidate enhancers provides a rich source for studying neocortex development and evolution.

Three major cell populations contribute critically to neocortex development at E14.5 (Figure 1A). By curating population specific gene expression data into a GREAT ontology, we show that enhancers serving all three major populations are enriched within our set. We also used other GREAT ontologies to subdivide the large enhancer mass into subsets that serve specific processes of interest in different dorsal cerebral wall populations at this stage, strongly suggesting that despite the heterogeneity of input material, numerous insights can be had into the different processes taking place in this developing tissue (Table 1).

Key transcription factors (TFs) often bind directly (both proximally and distally) next to a large number of genes in their relevant context [11]. This allows us to utilize motif discovery to predict key TFs and TF dimers found in a large number of our active enhancers (Figure 3). In circuit design terminology this property is known as large “fan out” (in this case of regulatory interactions) from TF to target genes (via binding sites and enhancers).

When we turn our point of view from regulators to regulated genes, we first looked for target genes with large “fan in”, namely genes in whose regulatory domains lie a larger than expected number of p300 peaks (Figure 4). The mammalian genome is known to contain multiple large gene deserts carrying numerous conserved and likely *cis*-regulatory sequences [34]. However, one cannot deduce from sequence patterns alone how many *cis*-regulatory regions are active simultaneously in any given functional context. Here we show that a number of genes carry dozens of p300 peaks in their regulatory domains during neocortex development, many more than would be expected by chance. It has been hypothesized that multiple seemingly-redundant enhancers co-exist in order to generate expression patterns that are robust to environmental variation [35], [36]. Multiple enhancers targeting the same gene also likely reduce the variability associated with stochastic gene regulation [37]. Finally, it is also possible that different enhancers target different cell populations during neocortex development. In focusing on the ten most heavily regulated genes (Table 2), we discover three well known genes in the context of neocortex development, and three additional genes already suspected of playing an important role because of their restricted expression pattern during neocortex development and correlations with neocortical-associated diseases. We also find two intriguing gene deserts, dense in p300 elements, that are flanked by two pairs of genes with no known role in neocortex development. In both cases, transcriptional evidence is not seen for other, possibly non-coding, transcripts within the gene deserts, and in both cases only one of the two flanking genes appears to be expressed in the neocortex (Figure 4). In both cases this gene is either a known transcription regulator (*Mn1*), or is suspected of being one (the coiled-coil *Gse1* gene).

Perhaps one of the most challenging questions to ask from enhancer data such as ours lies at the intersection of genomics and genetics. Namely, which enhancers form the “weak points” of the network, or in other words, which enhancers will cause a clear developmental defect when mutated? The *Fezf2* E4 enhancer provides one such example in the context of the neocortex (Figure 7). The *Fezf2* gene belongs to a small network of transcription factors that controls cell fate determination within the neocortex [38]. Scanning the p300 landscape around the other genes in this network we find a particularly compelling landscape around the *Tbr1* gene, with a single peak proximal to this key target gene, and few others further away (including elt4 from Figure 2, over 50 kb upstream). At the center of the proximal peak lies a co-opted instance of AmnSine1. Strikingly, AmnSine1 overlapping p300 peaks are found next to several additional key genes for early neocortex development, suggesting that perhaps a subset of AmnSine1 co-option events were crucial in laying out the cortical projection network as we know it today [39].

Members of multiple interspersed repeat families have likely contributed important enhancers during genome evolution (Figure 6). This contribution has been previously noted based on the large intersection between conserved non-coding sequence and sequences from mobile element origins [30]. The functional roles of the co-opted loci, however, could not be easily deduced from sequence alone. By intersecting mobile elements with functional data, we are able to assign specific functions to subsets of loci. This allows us to highlight several poorly studied repeat families in the context of neocortex development, as well as shed new light on cases such as the MER121 family, which was previously studied in sequence [40], but can now be implicated in contributing to limb development (Figure 6). Interestingly, nearly half of AmnSine1 and MER121 human instances were very recently found to overlap open chromatin from 41 cell types, suggesting possible enhancer activity in multiple additional contexts [41].

Two of our tested enhancers – elt4 and elt7 – drive expression in the most superficial cells of the developing neocortex (Figure 2). These patterns match a domain of the expression and functional activity of *Tbr1* and *Bhlhb5*, their nearby and likely respective target genes [42], [43]. The other six enhancers are active primarily in the CP and SVZ-IZ. In total, six of the eight positive enhancers drive expression largely within the domain of activity of the putative target gene [14], [44]. Two enhancers drive expression patterns that include a zone outside the detected expression regions of the putative target. These elements – elt1 and elt6 – drive expression in the CP and SVZ-IZ although their putative target genes (*Eomes*/*Tbr2* and *Id4*) are expressed primarily in the SVZ-IZ and VZ. These elements may regulate a different nearby gene or their *in vivo* expression pattern may be modified by flanking regulatory sequence or epigenetic state not captured in our transgenic constructs. Interestingly, our validated enhancers mostly drive expression outside the VZ. Our statistical analysis suggests that our full set is strongly enriched near genes expressed predominantly in the VZ (Table 1). Moreover, of 40 enhancers showing expression in the VZ of the dorsal pallium at E11.5 [15], 26 (65%) are marked by p300 peaks in our E14.5 set.

Finally, as the large (but far from exhaustive) number of vignettes in our paper illustrates, the biggest challenge for the study of functional genomic data is twofold: First, to develop a set of approaches and tools to mine these datasets and their combinations for the almost staggering wealth of information they offer. Second, a broader challenge relates to the coming together of different disciplines of researchers, including functional genomicists, computational biologists, developmental biologists, geneticists, and more, so that the mining of this data is maximized.

## Materials and Methods

### p300 ChIP-seq

Embryos were harvested from timed pregnant embryonic day 14.5 (E14.5) Swiss Webster mice (Charles River). The dermis, skull mesenchyme, and bone primordia were removed and cortical caps were dissected with curved forceps and placed in PNGM (Lonza). The medial structures, cortical hem/hippocampus and choroid plexus were cut off in a secondary excision. Dissected dorsal cerebral wall tissue (0.15 g) was snap frozen in liquid nitrogen. Tissue was fixed in 1% formaldehyde for 15 minutes. Chromatin was isolated, sheared and immunoprecipitation was performed using 30 micrograms of chromatin and 4 micrograms of anti-p300 antibody, C-20 (Santa Cruz SC-585; Genpathway). Chromatin from the same sample was processed for the input control. Library construction and sequencing was done using the Illumina GA II format (Illumina). This produced 17,460,074 uniquely mapped 36 bp reads for the treatment and 15,669,334 uniquely mapped reads for the input control.

### ChIP-seq peak calling

ChIP-seq reads were mapped to the mouse genome (UCSC mm9 assembly, NCBI MGSCv37) using ELAND, retaining only reads that map uniquely with 2 or fewer mismatches. Peaks were called using MACS [45] with the p300 ChIP-seq reads as the treatment file, input DNA reads as the control file, and the parameters “--nomodel, --shiftsize = 100, -g mm”. Peaks overlapped by an exon, within 2.5 kb of a transcription start site, or suspected in non-unique read mapping were removed. Exon and transcription start site annotation was obtained from the UCSC knownGene track (build 5) [46]. The median fold enrichment over input for our 6,629 peaks is 7.11 (and average 7.83).

### Functional and expression enrichment analysis with GREAT

To evaluate functional and expression enrichments, we used GREAT v2.0.0 [11] with the default association rule (1 kb+5 kb basal domain with up to 1 Mb extension and curated regulatory domains) and default significance thresholds (region-based binomial fold ≥2, region-based binomial FDR≤0.05, gene-based hypergeometric FDR≤0.05). A lower region-based binomial fold criterion was used for the MGI Expression ontology.

We evaluated specific enrichment in the ventricular zone, subventricular and intermediate zones, and cortical plate using a custom-built ontology based on a recent RNA-seq dataset [14]. We consider a gene to be specific to a layer if it has a layer RPKM (mean Reads Per exonic Kilobase per Million mapped reads) >64 and >2×(RPKM of the adjacent layer, or average of both adjacent layers for the subventricular and intermediate zones).

### Mouse transient transgenic enhancer assay, transfections, and sectioning

The ten candidate elements for transgenic and transfection assays had p300 fold enrichments ranging from 4.92 to 19.18 (90th to 1st percentile, with average rank in the 37th percentile). Candidates were PCR amplified from mouse genomic DNA (Clontech), cloned into pENTR/D (Invitrogen), and then Gateway cloned with LR Clonase (Invitrogen) into a HSP68-lacZ-Gateway DEST vector (a gift from Nadav Ahituv, UCSF). Primers are listed in Table S2.

Constructs were linearized with SalI prior to injection. Transgenic mice were generated by pronuclear injections of FVB embryos (Xenogen Biosciences, Cranberry, NJ). Embryos were harvested at embryonic day 14.5, fixed, whole mount stained for lacZ, embedded in paraffin, sectioned, and counterstained using Nuclear Fast Red (Vector Laboratories).

For transfection of cortical neurons, elements were cloned into the firefly luciferase vector, pGL4.23 (Promega) containing Gateway cassette A (Invitrogen). Neurons from the dorsal cerebral wall were dissected as for ChIP-seq, dissociated using 0.25% trypsin and 10 ug/ul DNase, transfected with experimental luciferase construct and a pRLTK Renilla control in a 96-well nucleoporator (Lonza) then plated onto poly-D-lysine coated 96-well plates (NUNC) in PNGM (Lonza). Media was changed 4–6 h after transfection, and luciferase assays were done 48 h after transfection. Luciferase assays were done using a DLR 100 kit (Promega) according to the manufacturer's instructions and read using a Promega Glomax luminometer.

### Ethics

All animals were treated under protocols #18487 and #21758 approved by Stanford University Institutional Animal Use and Care Committee.

### Motif discovery and enrichment analysis

Length and GC-matched regions were selected randomly from the mouse genome to provide a null set for the 6,629 E14.5 peaks. We then ran ten different published motif discovery tools on the set of peaks and controls: Allegro [47], AlignAce [48], BioProspetor [49], CisFinder [50], MDscan [51], MEME [52], MoAn [53], MotifSampler [54], NestedMica [55], and Weeder [56]. Near identical motif predictions were combined. In a previous work we compiled a library of motifs (position weight matrices) for hundreds of different transcription factors from public motif databases and primary literature [57]. We combined the *de novo* motif candidates with our library of known motifs. The set of known and putative novel motifs was then predicted at a motif match threshold of 0.9 [58] in both our peaks and the control set of regions. Motif fold enrichment was calculated as the number of candidate enhancers with a match to the motif divided by the number of random regions with a motif match. Motifs over two fold enrichment are reported in Figure 3.

### Evolutionary conservation analysis

We considered a candidate enhancer to be under purifying selection if it overlaps a region from the UCSC mm9 PhastCons Elements track (phastConsElements30way) that scores at least 350 [59]. We tagged candidates with depth of conservation based on pairwise alignment nets from UCSC [60]. We obtained all regions of the genome in the level 1 and 2 nets; eliminated large duplications (genomicSuperDups track) [61], pseudogenes (pseudoYale60), and known exons (knownGene:exon) [46]; and considered a basepair reliably conserved to a given clade only if it is conserved to the previous clade. Clades were represented by: euarchontoglires (human hg19, chimp panTro3, rhesus rheMac2); eutheria (elephant loxAfr3); mammalia (platypus ornAna1); amniota (chicken galGal3, lizard anoCar2); tetrapoda (frog xenTro3); gnathostomata (tetraodon tetNig2, fugu fr2, zebrafish danRer7, stickleback gasAcu1, medaka oryLat2). For clades with multiple representatives, a basepair is considered conserved if it aligns to any of the representatives, except two genomes are required for gnathostomata. A candidate enhancer is tagged with the deepest clade to which at least 200 bp of the candidate is conserved.

In Figure 5B, “non-exonic basepairs” are all basepairs in the mouse genome not in large duplications, pseudogenes, exons, or gaps.

### Overlap with VISTA Enhancer Browser enhancers

The VISTA Enhancer Browser [23] includes results for mouse transgenic enhancer assays for candidate human DNA sequences. We obtained 1,255 tested human sequences, and mapped the sequences to the mouse genome (mm9 assembly) using liftOver (-minMatch = 0.8) and lastz (--seed = match6, --hsptresh = 1800, --gappedthresh = 5000, sequence identify ≥65%, entropy ≥1.8). We successfully mapped 1,188 enhancers, including 176 forebrain enhancers. The tested sequences overlap 214 of our candidate enhancers, with 93 active in the forebrain. The significance of tested E14.5 candidate enhancers driving activity in the different mouse tissues (Figure 5C) is calculated using a hypergeometric enrichment test (for example, forebrain: hyper[93/214; 176/1,188]).

### Overlap with zebrafish cneBrowser enhancers

The zebrafish cneBrowser [24], [62] includes results for zebrafish transgenic enhancer assays for candidate zebrafish DNA sequences. We obtained 164 tested zebrafish sequences, and mapped the sequences to the mouse genome (mm9 assembly) using lastz (--seed = match6, --hsptresh = 1800, --gappedthresh = 5000, sequence identify ≥65%, entropy ≥1.8). We successfully mapped 129 enhancers (21 overlap a candidate E14.5 enhancer), including 31 forebrain enhancers (11 overlap). The significance of tested candidate E14.5 enhancers driving activity in zebrafish tissues (Figure 5D) is calculated using a hypergeometric enrichment test (for example, forebrain: hyper[11/21; 31/129]).

### Overlap with mobile elements

The repeat-annotations (RepeatMasker open-3.2.8) for the mouse genome (mm9) were downloaded from RepeatMasker (http://www.repeatmasker.org/). For each p300 ChIP-seq set, we measured the observed overlap with each interspersed repeat family. To determine the expected overlap, our p300 set was shuffled randomly across the genome 10,000 times. For each of these shuffles, the overlap with each repeat family was measured. The expected overlap is the average of these shuffles. Fold enrichment is calculated as observed/expected. The Z-score is (observed-expected)/standard deviation. Note that because we used only uniquely mapped reads (of length 36) we may miss some peaks and overlaps with the most recently active repeat families whose genomic copies may still hold long stretches of identical bases. However, all families highlighted in the text are old and no longer active such that the reads overlapping them resolve accurately and comprehensively.

## Supporting Information

Figure S1 All whole mounts of transgenic embryos for enhancer elt1 (Figure 2A), near *Eomes*.
(TIFF)

Figure S2 All whole mounts of transgenic embryos for enhancer elt2 (Figure 2B), near *Satb2*.
(TIFF)

Figure S3 All whole mounts of transgenic embryos for enhancer elt3 (Figure 2C), near *Neurod2*.
(TIFF)

Figure S4 All whole mounts of transgenic embryos for enhancer elt4 (Figure 2D), near *Tbr1*.
(TIFF)

Figure S5 All whole mounts of transgenic embryos for enhancer elt5 (Figure 2E), near *Auts2*.
(TIFF)

Figure S6 All whole mounts of transgenic embryos for enhancer elt6 (Figure 2F), near *Id4*.
(TIFF)

Figure S7 All whole mounts of transgenic embryos for enhancer elt7 (Figure 2G), near *Bhlhb5*.
(TIFF)

Figure S8 All whole mounts of transgenic embryos for enhancer elt8 (Figure 2H), near *Auts2*.
(TIFF)

Table S1 The mouse mm9 coordinates of 6,629 p300 peaks obtained from E14.5 dorsal cerebral wall ChIP-seq.
(XLSX)

Table S2 PCR primers used to clone candidate enhancer elements from mouse genomic DNA. 5′-CACC” was added to each left primer for cloning into pENTR/D.
(DOCX)
