# Supplementary figures and images for: The Enhancer Landscape during Early Neocortical Development Reveals Patterns of Dense Regulation and Co-option
Source: PLoS Genet. 2013 Aug 29;9(8):e1003728. doi: 10.1371/journal.pgen.1003728 (PMC3757057; doi:10.1371/journal.pgen.1003728)

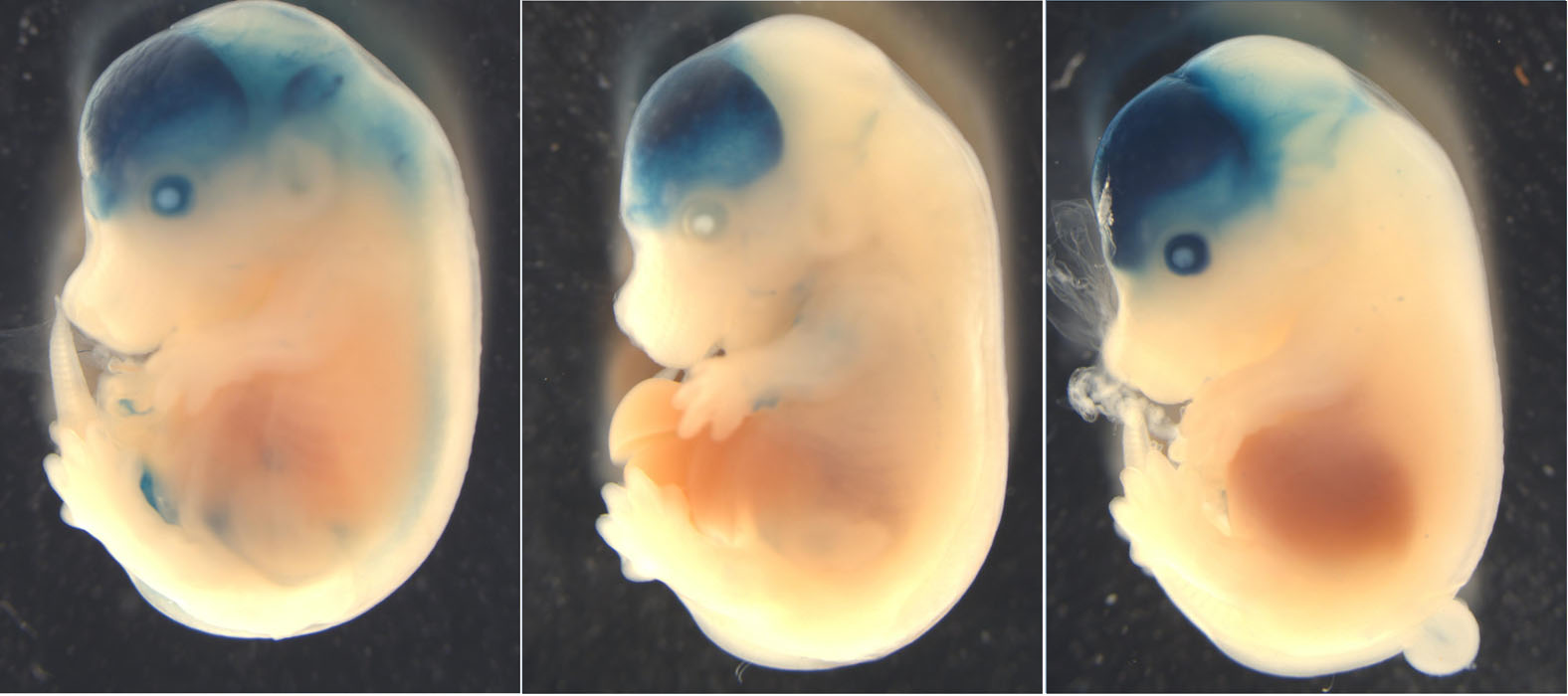

Supplement: Figure S1 — All whole mounts of transgenic embryos for enhancer elt1 (Figure 2A), near Eomes. (TIFF) [file pgen.1003728.s001.tiff]

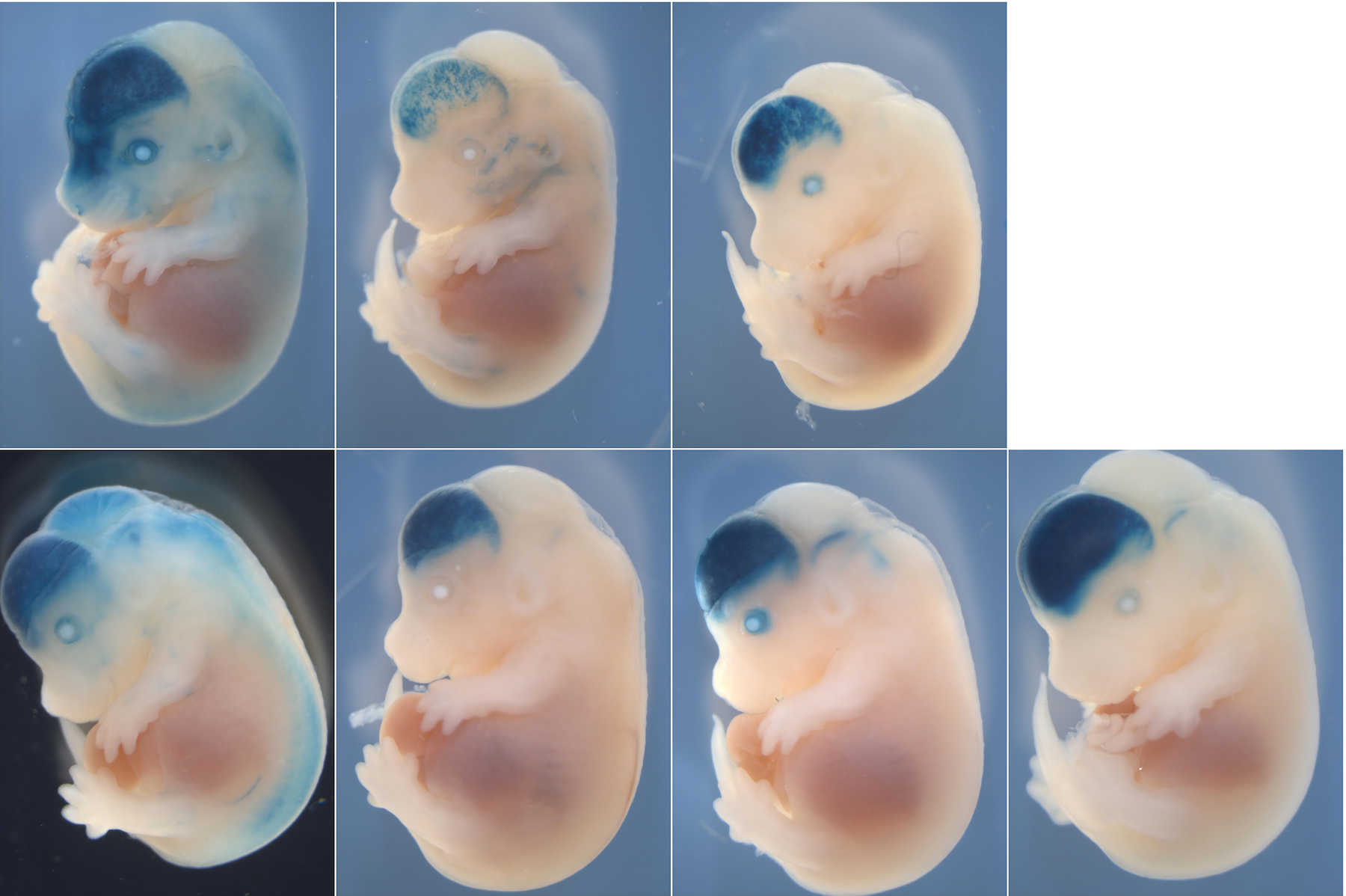

Supplement: Figure S2 — All whole mounts of transgenic embryos for enhancer elt2 (Figure 2B), near Satb2. (TIFF) [file pgen.1003728.s002.tiff]

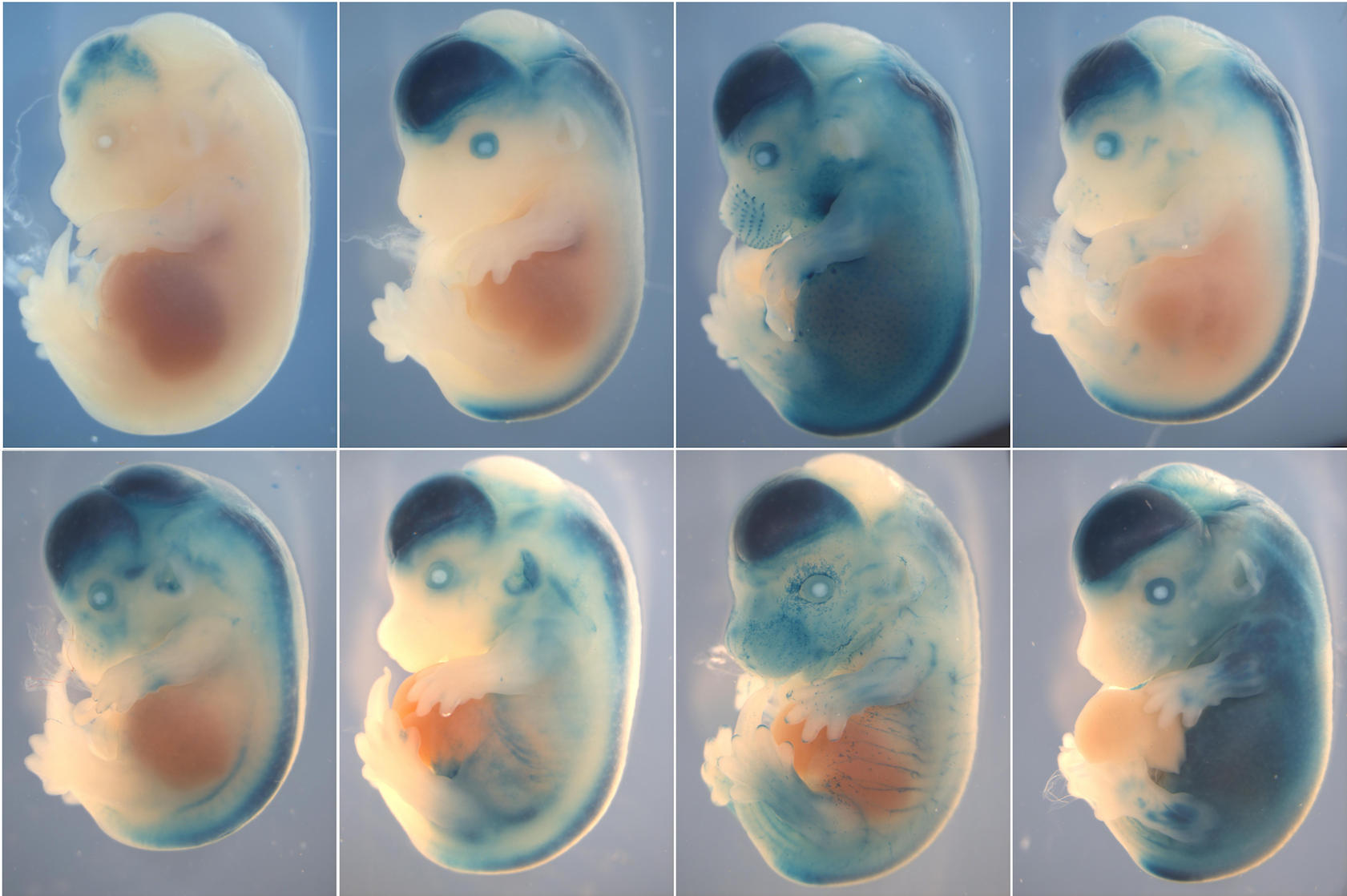

Supplement: Figure S3 — All whole mounts of transgenic embryos for enhancer elt3 (Figure 2C), near Neurod2. (TIFF) [file pgen.1003728.s003.tiff]

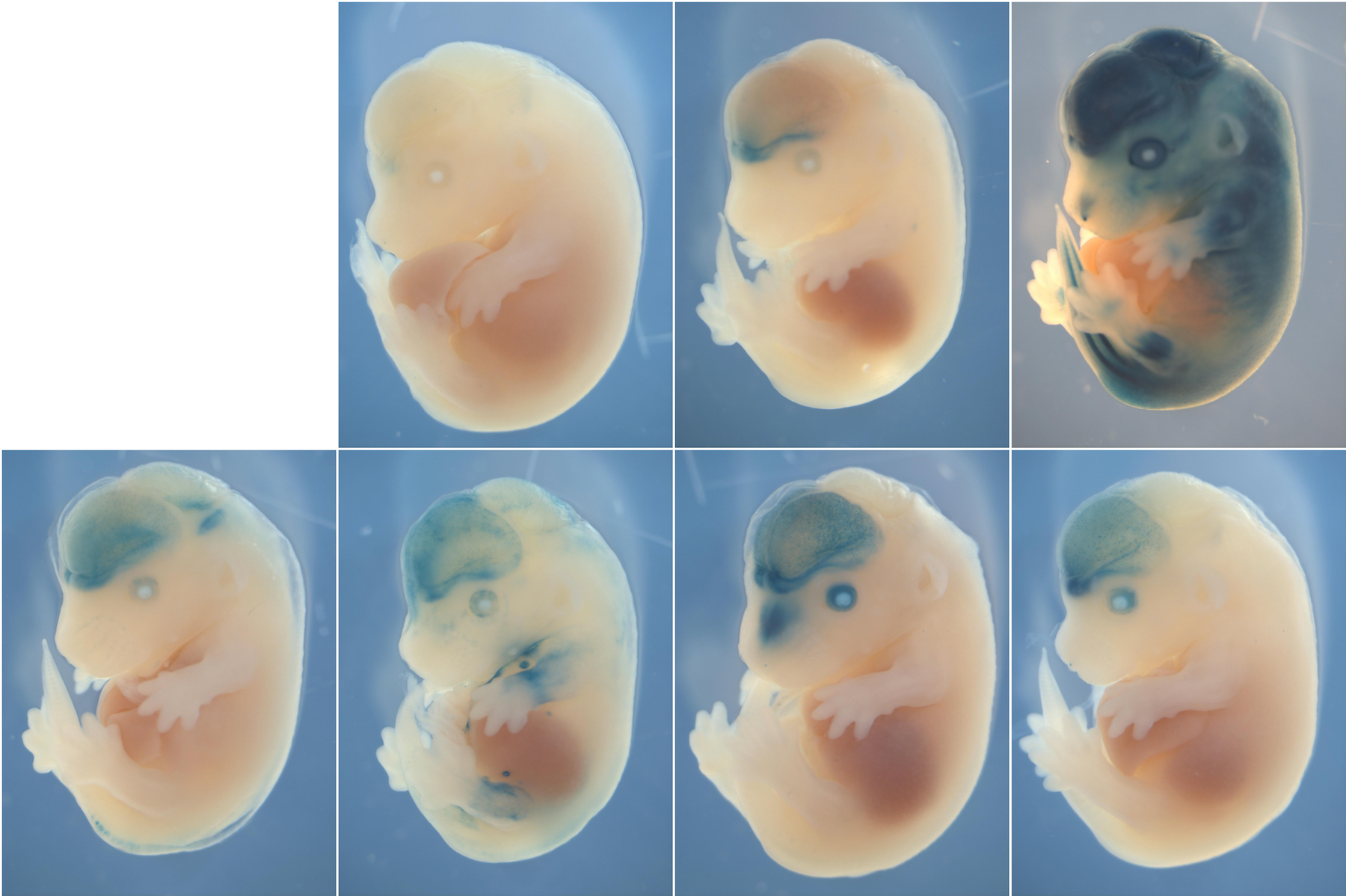

Supplement: Figure S4 — All whole mounts of transgenic embryos for enhancer elt4 (Figure 2D), near Tbr1. (TIFF) [file pgen.1003728.s004.tiff]

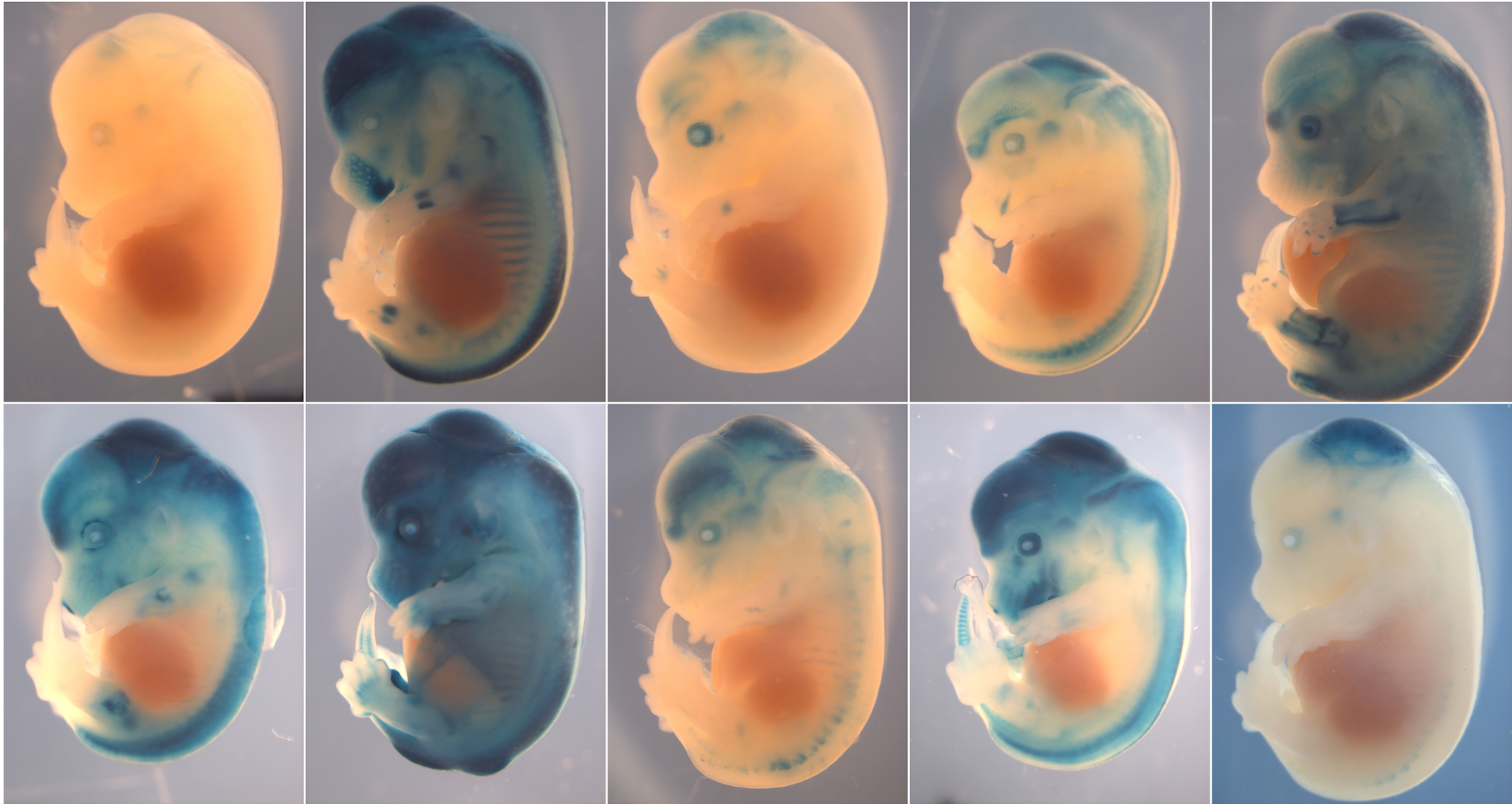

Supplement: Figure S5 — All whole mounts of transgenic embryos for enhancer elt5 (Figure 2E), near Auts2. (TIFF) [file pgen.1003728.s005.tiff]

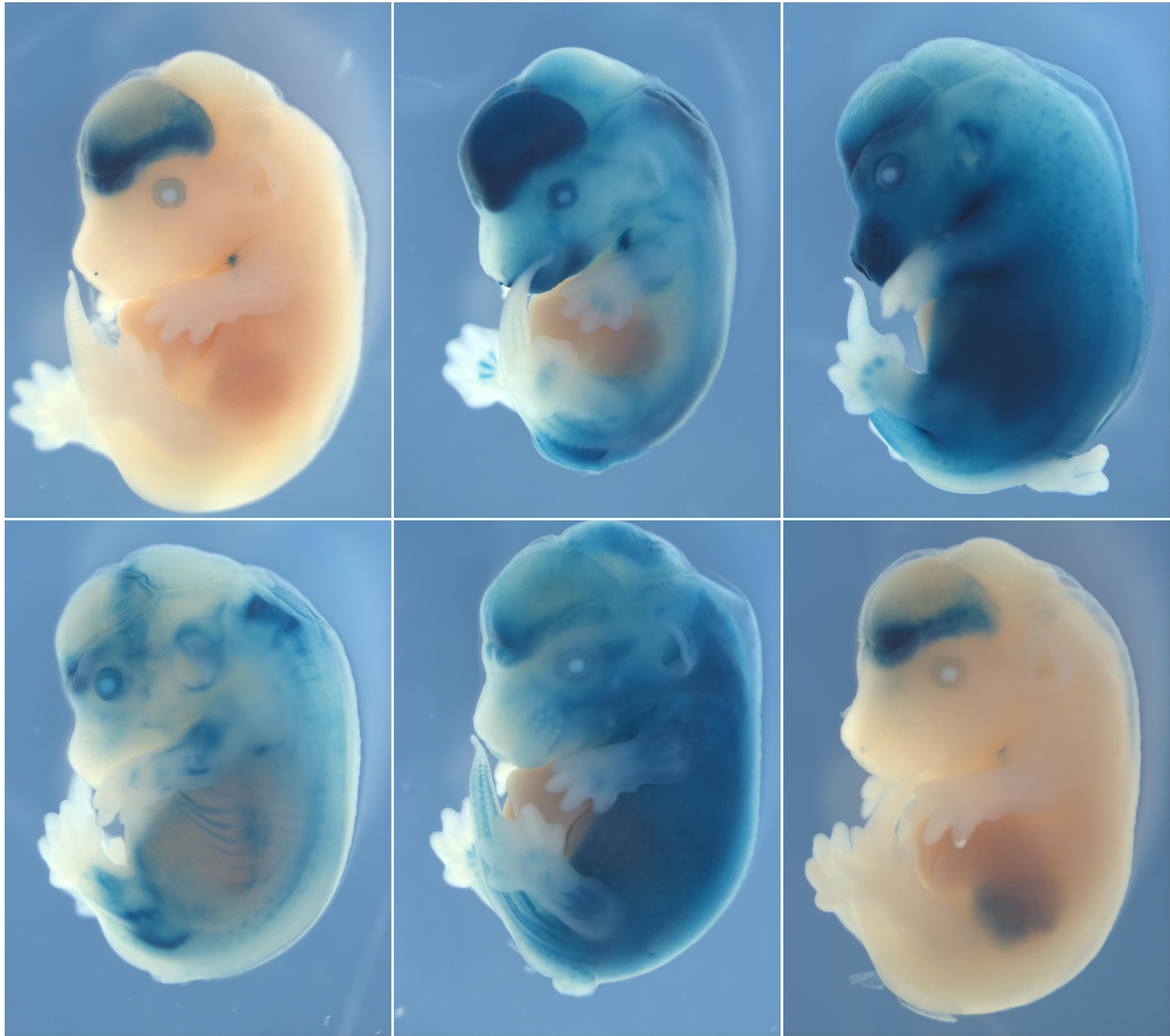

Supplement: Figure S6 — All whole mounts of transgenic embryos for enhancer elt6 (Figure 2F), near Id4. (TIFF) [file pgen.1003728.s006.tiff]

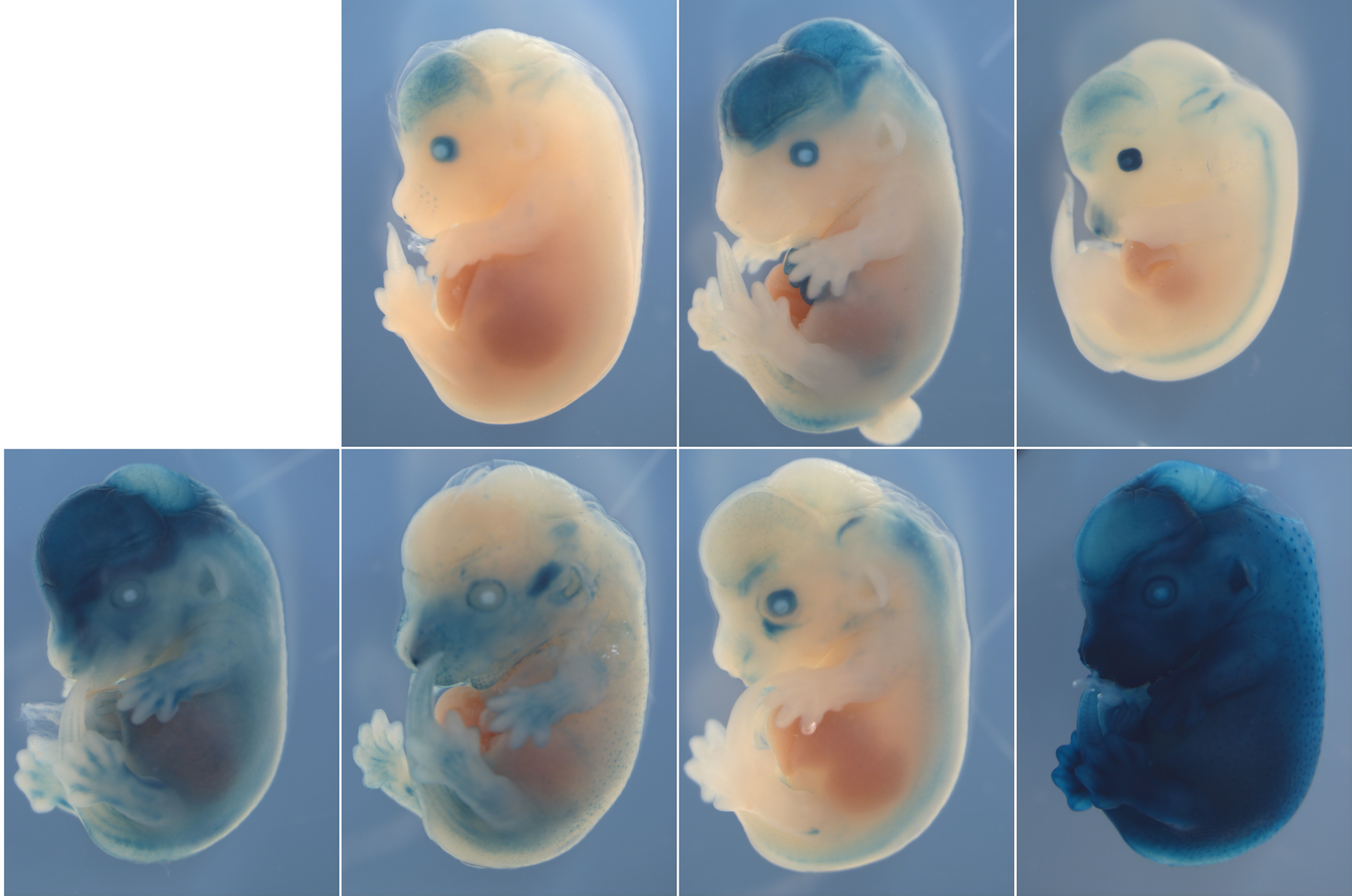

Supplement: Figure S7 — All whole mounts of transgenic embryos for enhancer elt7 (Figure 2G), near Bhlhb5. (TIFF) [file pgen.1003728.s007.tiff]

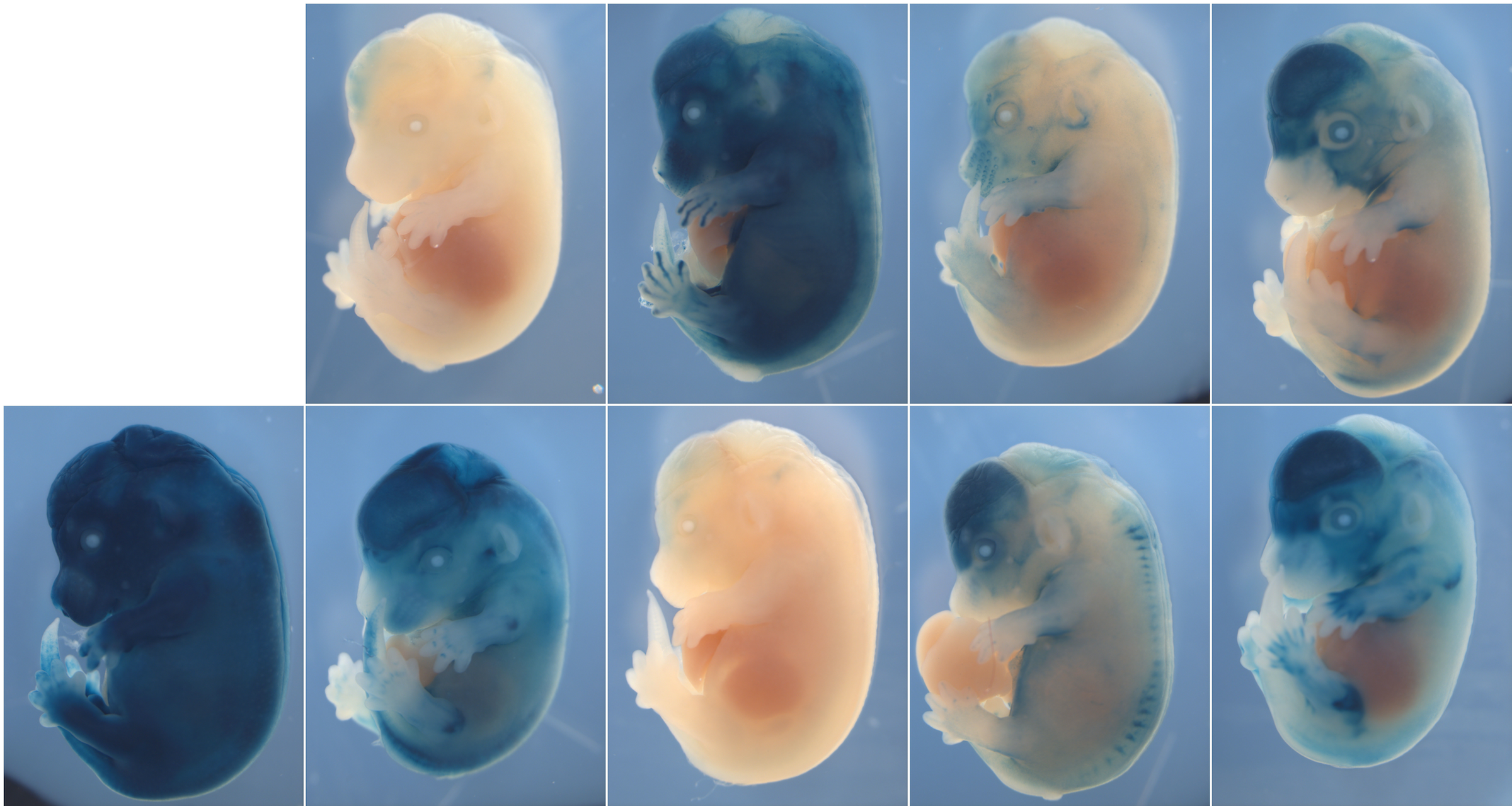

Supplement: Figure S8 — All whole mounts of transgenic embryos for enhancer elt8 (Figure 2H), near Auts2. (TIFF) [file pgen.1003728.s008.tiff]
