# Supplementary material for: The Enhancer Landscape during Early Neocortical Development Reveals Patterns of Dense Regulation and Co-option
Source: PLoS Genet. 2013 Aug 29;9(8):e1003728. doi: 10.1371/journal.pgen.1003728 (PMC3757057; doi:10.1371/journal.pgen.1003728)
Supplement: Table S2 — PCR primers used to clone candidate enhancer elements from mouse genomic DNA. 5′-CACC” was added to each left primer for cloning into pENTR/D. (DOCX) [file pgen.1003728.s010.docx]

| **Element Name** | **Forward primer** | **Reverse primer** |
| --- | --- | --- |
| elt1 | AACCCCGCTGTCTTCTAGGT | TTTTTAACAATGTTCACACACTTCA |
| elt2 | GCGTTCACTTTCCCAGCTAC | CCTGATAAATGCTGAGGCTAGA |
| elt3 | TGGTTGGAAGTTAGCTCTGATG | AAGAGACTGGGGTTGGAGGT |
| elt4 | ATTCCGGAGCCCAGAAGTAT | CATGAAACCCTTCAAAATTGC |
| elt5 | GTCCACGGAGACCAGAGGTA | CATGCACATGCACAAGAACA |
| elt6 | GCATGCTTGTGCTATCCATT | CCCATCATGCATTCTGCTAA |
| elt7 | TGGACTTCGAATAATGAGGACA | TGTTTTCTCTGGCTGTCTCAAA |
| elt8 | TGTCAAAGGCAATTAATGAGAAAA | GAGATGGTTGCTTCATTGCAT |
| elt9 | CCTTCATCTGTTTCTCTTAATTAGCC | GGGGGAGGGAGGTTGTAGT |
| elt10 | GCACCTGGATCCCACATC | GAGTCAGTCTGTCCAAGTAAGCTG |

Table S2
